# Left atrial volume quantification by transthoracic echocardiography versus cardiovascular magnetic resonance: a systematic review and meta-analysis

**DOI:** 10.1007/s10554-025-03455-1

**Published:** 2025-07-23

**Authors:** Masliza Mahmod, Sacha Bull, Tushy Kailayanathan, Tom A. Davis, Alessandra Borlotti, Iulia A. Popescu, Indrajeet Das, Malgorzata Wamil, Helena Thomaides Brears, Rajarshi Banerjee, Amitava Banerjee

**Affiliations:** 1 Perspectum Ltd, Oxford, UK; 2 Division of Cardiovascular Medicine, Radcliffe Department of Medicine, University of Oxford, Oxford, UK; 3 Department of Cardiology, University, Royal Berkshire Hospital NHS Foundation Trust, Reading, UK; 4 Cardiothoracic Imaging, University Hospitals of Leicester NHS Foundation Trust, Leicester, UK; 5 Department of Cardiology, Mayo Clinic Healthcare in London, London, UK; 6 Nuffield Department of Reproductive and Women’s Health, University of Oxford, Oxford, UK; 7 Institute of Health Informatics, University College London, London, UK; 8 Department of Cardiology, University College London Hospitals NHS Foundation Trust, London, UK; 9 Medical Lead, Cardiovascular Clinical Science, Perspectum Ltd, Oxford, UK

**Keywords:** Left atrial volume, Heart failure, Echocardiography, Magnetic resonance, Diagnostic accuracy, Heart failure preserved ejection fraction

## Abstract

Heart failure (HF) with preserved ejection fraction (HFpEF) is diagnosed by symptoms and/ or markers of congestion with cardiac dysfunction despite ‘normal’ (preserved) left ventricular (LV) EF. Of the criteria, left atrial volume (LAV) is being increasingly used, with cardiovascular magnetic resonance (CMR) recognised as the gold standard for volumetric assessment. We searched databases for studies with LAV and indexed (LAVi) measurements obtained through both transthoracic echocardiography (TTE) and CMR. We identified 17 articles, encompassing 1203 individuals with cardiac disease. TTE showed more frequent measurement failure (6% [95% CI 2, 11]) compared to CMR (1% [0, 5]). TTE underestimated values compared to CMR, with a bias of -20mL [95% CI -30, -11] for LAV and − 9 mL/m^2^ [95% CI -13, -5] for LAVi (*p* < 0.001). TTE misclassified LA enlargement in 38% [95%CI 25, 52) of cases diagnosed by CMR. TTE systematically underestimates LAV and should be used with caution.

**Graphical abstract:**

Quantification of left atrial enlargement. Left atrial volume (LAV) enlargement is one of the imaging criteria to diagnose heart failure with preserved ejection fraction (HFpEF). While cardiovascular magnetic resonance (CMR) is recognised as the gold standard technique, transthoracic echocardiography (TTE) is routinely used to measure LAV. This review identified 17 articles, encompassing 1203 individuals who had LAV and/or indexed (LAVi) measurements obtained from both TTE and CMR. When compared to CMR, TTE showed higher failure rates (6% vs 1%), underestimated LA values, and misclassified LAV enlargement in 38% of cases diagnosed by CMR.

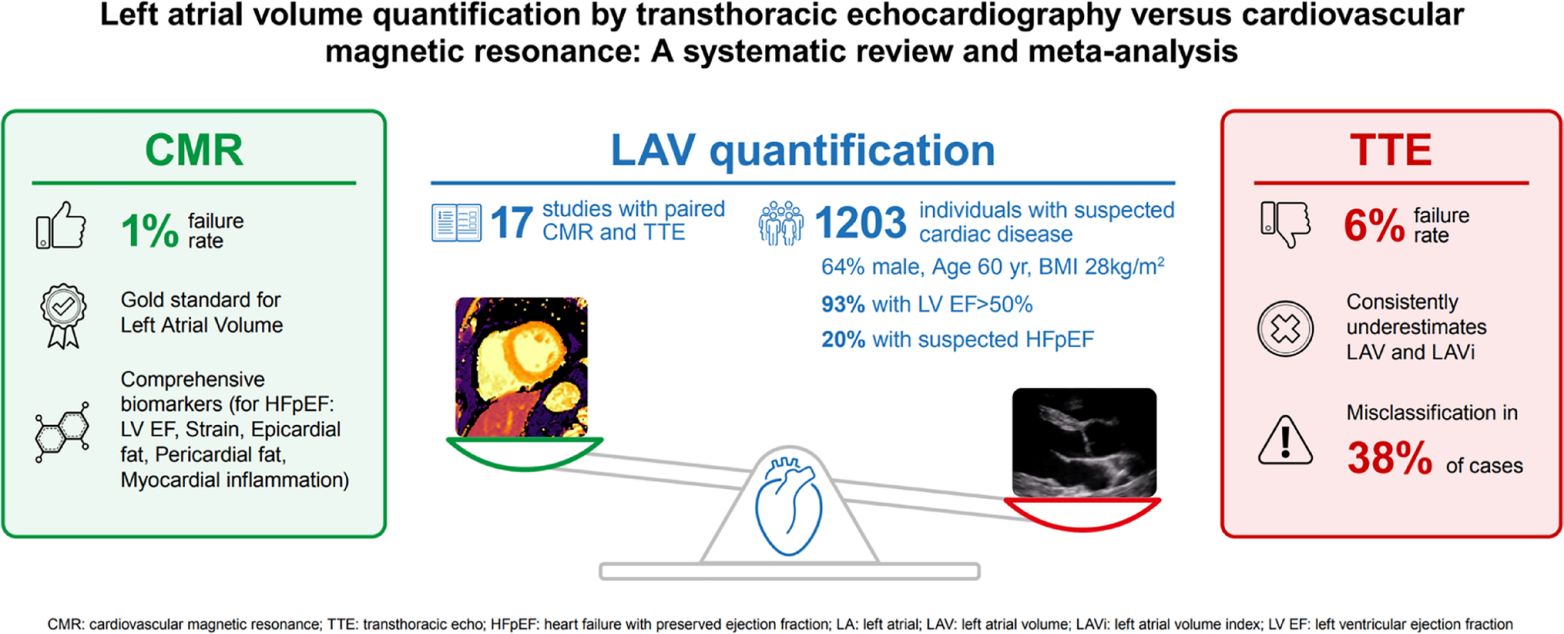

**Supplementary Information:**

The online version contains supplementary material available at 10.1007/s10554-025-03455-1.

## Introduction

Heart failure (HF) with preserved ejection fraction (HFpEF) is a clinical syndrome in which patients exhibit signs and symptoms of HF despite normal or near normal left ventricular ejection fraction (LVEF) [1]. HFpEF currently represents over half of newly diagnosed cases of HF in the community, and, with an incidence rate of 27 cases per 10,000 person years, will affect 1 out of 10 adults in their lifetime [2]. Nevertheless, HFpEF is still underdiagnosed in up to 64% of cases due to lack of awareness, continued diagnostic uncertainty, and absence of a consensus on the best clinical pathway [3–5]. This delays therapeutic intervention and development of new drug therapies.

Diagnosis of HFpEF requires symptoms and/or signs of pulmonary congestion supported by objective evidence of preserved LVEF (> 50%) and diastolic dysfunction or raised filling pressures [6, 7]. The imaging biomarkers of HFpEF are increased left atrial volume (LAV), LV mass and/or impaired LV strain [6–8]. These imaging metrics provide phenotypic, diagnostic and prognostic value in HFpEF and other cardiovascular conditions and outcomes [5, 9–13]. Elevation of the blood biomarker N-terminal pro B-type natriuretic peptide (NT-proBNP) indicates congestion.

Of the imaging criteria, LAV is routinely used in HFpEF. Importantly, LAV indexed to body surface area (BSA)—LAVi is an indirect measure of LV filling pressures [14] and correlates with indices of LV diastolic function [15]. When compared to LA diameter or area, LAVi is a more reliable marker of chronic LA remodelling [16, 17]. The European Society of Cardiology Guidelines state that LAV index (LAVi) > 34 mL/m^2^, in sinus rhythm and in absence of atrial fibrillation (AF), indicates disease and significant LA dilatation in HFpEF [18]. Values above that threshold have been associated with death, HF, AF, and ischaemic stroke outcomes [19]. Cardiovascular magnetic resonance (CMR) has superior spatial resolution allowing more accurate and reproducible cardiac chamber quantification and is the gold standard for imaging cardiac structures and assessing cardiac volumes [20–22]. Nevertheless, transthoracic echo (TTE) is often the first line imaging modality due to high availability, although TTE sensitivity varies substantially (25–80%) [23]. Therefore comparing CMR to TTE is relevant in clinical setting and HFpEF research.

To ensure a standardised and scalable imaging approach is implemented in clinical trials and studies for HFpEF, we undertook a systematic review and meta-analysis to explore LAV quantification measured by CMR and TTE. In this head-to-head comparison of the two techniques in HFpEF and other cardiac disease cohorts, we aimed to compare: [1] technical performance for measurement of LA enlargement [2], LAV values and [3] classification of LA enlargement and prognostic accuracy.

## Methods

### Data sources, search strategy

This systematic review and meta-analysis was conducted following the Preferred Reporting Items for Systematic Reviews and Meta-Analyses (PRISMA) guidelines [24].

The following keywords were used for the final search: “cardiovascular magnetic resonance imaging/CMR”, “echocardiography/echo/transthoracic echo/TTE”, “left atrial volume/LA size”, “reproducibility”, “repeatability” and “diagnostic accuracy”. The literature search was performed on 8th November 2024 on PubMed for abstracts published between January 2012 to November 2024. The search was restricted to human studies in English. Inclusion of “HFpEF/heart failure/HFA-PEFF” in the key words did not identify any additional studies. Additional studies known to the cardiologist co-authors were included as grey literature.

### Study selection and eligibility criteria

Studies with CMR and 2D TTE on the same subjects to derive LAV measurements with enough data for qualitative and quantitative analysis were selected. Two authors screened the abstracts and titles and 1 author retrieved and screened full texts for eligibility. Eligibility of full text selection was finalised by consensus agreement between co-authors to ensure a minimum dataset of paired LAV measurement by 2D TTE and CMR. Only articles in English were considered. The PICOS criteria (population, intervention, comparison, outcome and study type) were: P: adult patients (≥ 18 years old) with or suspected cardiac disease; I: CMR; C: 2D transthoracic echocardiography; O: LAV measurement; S: original research but not literature reviews. Articles only reporting 3DE TTE or transoesophageal echocardiography were excluded. Due to the paucity of articles solely on HFpEF, studies on other cardiac diseases were included.

### Data extraction and quality assessment

Five authors extracted the following data from the included studies: PMID, author’s last name, publication year, country, sample size, study design, methodology for CMR and TTE, population characteristics and demographics, LAV, measurement bias between TTE and CMR LAV, LV EF, technical performance, diagnostic accuracy, prognostic value, agreement/correlations between LAV values CMR vs. TTE. LAV measurements included the maximum LAV that is measured just before the opening of the mitral valve (LAV_max_) and or the LAV indexed to body surface area (BSA) (LAVi). Measurement difference is defined as the patient-by-patient difference in the LAV measurement by CMR vs. TTE derived from Bland-Altman analysis. Technical performance for LAV included: the number of measurement failures (measurement failure rate), the variability in measurement following re-analysis of the same scan images by a second operator (inter-operator variability) or by the same operator after a time interval (intra-operator variability), scan-rescan repeatability (the difference in the measurement when the same individual is re-scanned with the same device under identical conditions), manufacturer reproducibility (the difference in the measurement when the same individual is scanned with the same devices from different manufacturers).

Two authors independently assessed the quality of the studies included in the primary meta-analysis using a modified version of the Newcastle–Ottawa quality assessment scale [25]. When there were different assessments, discussion was made in detail until consensus was achieved with input from an additional author. Funnel plots and Egger’s tests were not performed because the number of studies examined in each case was below the minimum required to sufficiently power these tests.

### Statistical analysis

We looked at the measurement failure rate of TTE and of CMR for LAV (when a measurement was not reported) and the misclassification rate (when CMR diagnosed LA enlargement and TTE classified the LAV as normal). To perform statistical tests, varying forms of averages, and their associated dispersion, were converted to mean and standard deviation [26]. Where applicable, values were transformed and fixed effects or random effects models were utilised to provide pooled estimates across studies, alongside Cochran’s Q and I-squared for tests of heterogeneity. All statistical analyses were performed in R Version 4.4.2.

## Results

### Literature search results

After conducting an electronic search on PubMed/EMBASE and consultation with experts in the field, we obtained a total of 131 articles without duplicates. 87 irrelevant articles were excluded based on screening of the title and abstract. 44 articles required full-text screening, 1 was not accessible even after contacting the authors, and 17 studies were included in both the qualitative, and quantitative analysis, as shown in Supplementary Fig. 1.

### Summary of the included studies

The 17 studies yielded 1203 participants in which LAV or LAVI were measured by both 2D TTE and CMR (from 1482 with TTE and 1288 with CMR). The baseline data of these studies are presented in Table 1, alongside the summary of the included studies in Supplementary Table 1. On average, where reported, participants were aged 60 years, male participants represented 64% of total and median BMI was 28 kg/m^2^. 45% of participants had hypertension and 16% had diabetes.

**Table 1:** Characteristics of selected studies

Author, year	Indication (*n*)	Age (years)^a^	% Male	% Hypertensive	% Diabetic	BMI (kg/m^2^)^a^	BSA (m^2^)^a^	LV EF (%)^a^
Backhaus SJ, 2023 [33]	HFpEF (by RHC or stress TTE) (34)	69 (67–77)	26	79	15	29 (27–33)	NR	59 (55–65)
	Dyspnoea (34)	66 (52–73)	44	79	15	28 (25–32)	NR	58 (53–62)
Ng MY, 2023 [53]	HFpEF (by RHC) (53)	78 (74–82)	40	89	42	26 (24–29)	NR	63 (56–66)
	Dyspnoea (38)	70 (64–76)	45	74	32	27 (24–32)	NR	63 (60–68)
	Healthy (17)	64 (60–67)	35	6	0	21 (20–24)	NR	65 (60–68)
Rahi W, 2024 [54]	HFpEF (by RHC, PCWP > 15 mmHg) (33)	51 ± 15	39	55	27	30 ± 11	NR	66 ± 9
	Dyspnoea (RHC, PCWP < = 15 mmHg) (46)	60 ± 13	43	46	15	28 ± 10	NR	60 ± 9
Agner BF, 2014 [55]	Atrial fibrillation (34)	69 ± 6	65	50	62	NR	2.0 ± 0.2	NR
Buechel RR, 2013 [29]	Atrial fibrillation (60)	61 ± 10 [32–77]	70	57	5	27 ± 5 [19,40]	NR	55 ± 8 [35,75]
Rabbat MG, 2015 [56]	Atrial fibrillation (250 for CMR, 171 for TTE)	58 ± 10	72	57	13	29 ± 5	NR	NR
Chung, 2021 [57]	HCM, Sarcomere+ (67 for TTE, 42 for CMR)	55 ± 14	63	42	18	NR	NR	64 ± 7
	HCM, Sarcomere- (145 for TTE, 93 for CMR)	61 ± 13	74	63	19	NR	NR	65 ± 6
	Age-, sex-matched controls (30 for TTE only)	60 ± 3	77	37	17	NR	NR	66 ± 4
Fujikura K, 2024 [32]	HCM (87)	45 ± 18	52	29	9	NR	1.9 ± 0.3	NR
Ricci F, 2019 [58]	HCM (Increased LAP, grade II-III) (23)	57 ± 11	78	43	13	29 (6)	2.0 (0.4)	75 ± 8
	HCM (Normal LAP, grade I) (46)	58 ± 11	85	39	20	29 (8)	2.0 (0.3)	72 ± 9
Isaac M, 2024 [59]	Stroke (44)	60 [25, 90]	59	NR	NR	NR	2.1 [1.5, 2.6]	NR
Dell’Aquila AM, 2012 [60]	Post-heart transplant (standard technique) (16)	48 ± 16	94	NR	NR	NR	NR	66 ± 7
Dell’Aquila AM, 2012 [60]	Post-heart transplant (bicaval technique) (19)	55 ± 10	95	NR	NR	NR	NR	70 ± 6
Zhu S, 2020 [28]	Post-heart transplant (31)	46 ± 14	74	NR	NR	23	1.7 ± 0.2	NR
Kühl JT, 2012 [61]	Post-STEMI (54 for CMR, 48 for TTE)	61 ± 10	76	28	4	28 ± 5	2.1 ± 0.2	56 ± 10
Florescu DR, 2021 [27]	Non-disease specific (198 for TTE)	67 (49–77)	64	12	NR	NR	1.8 [0.7, 2.0]	NR
	Non-disease specific (26 paired for CMR)	59 (17–83)	81	12	NR	NR	NR	NR
Mor-Avi V, 2012 [21]	Non-disease specific (92)	48 ± 18	62	NR	NR	NR	1.7 ± 0.3	NR
Perez de Isla L, 2014 [30]	Non-disease specific (70)	56 ± 18	60	29	20	NR	1.7 ± 0.3	42 ± 17
Ramos JG, 2020 [44]	Non-disease specific (46)	59 (46–68)	67	NR	NR	26 ± 4	2.0 ± 0.2	58 (50–62)

^a^Data are shown as mean ± SD, median (interquartile range), or median [range]

Only two studies were specifically on suspected HFpEF and a third described undifferentiated dyspnoea. The remaining studies included three on patients with atrial fibrillation (AF), three on patients with hypertrophic cardiomyopathy (HCM), one on patients with stroke, one on ST segment elevation myocardial infarction (STEMI) patients, two on heart transplant patients and 4 studies evaluating cardiology patients without specified indications. On average 93% of participants had LV EF > 50% in the 12/17 studies where it was reported.

The time interval between 2D TTE and CMR was reported in 12/17 studies and was within 7 days in all studies and within 1 day in 9/17. LAV processing by 2D TTE was biplanar in all 17 studies and the CMR measurement was also biplanar in 5/17 studies. CMR was either by multi-slice LA stack in the remaining studies (11/17) or single slice AL. The quality of the studies was good overall and there was low risk of bias (Supplementary Table 2).

### Technical performance

**Fig. 1:**
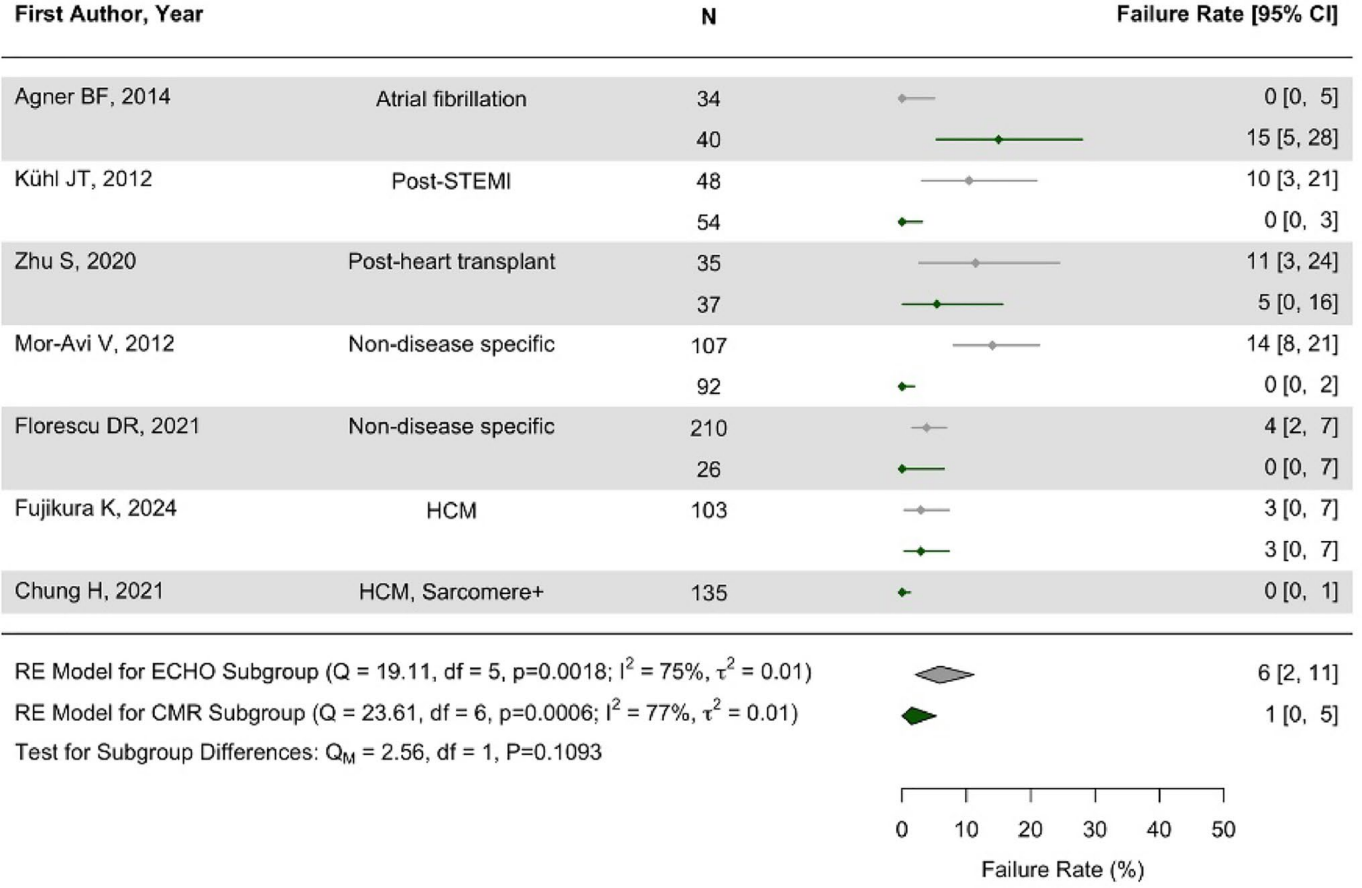
Measurement failure by CMR (green) compared to TTE (grey)

Six studies reported on measurement failure (images not analysable) from both CMR and TTE, and 1 study on measurement failure by CMR only (Fig. 1, Supplementary Table 3). TTE failed to provide a measurement more frequently (median 7% scan fails, pooled mean 6% [2, 11]) than by CMR (median 0%, pooled mean 1% [0, 5]). By CMR, 4/7 studies reported no failures, whereas by TTE only 1/6 study had no failures. Most frequent cause of measurement failures was poor image quality by TTE and patient compliance (claustrophobia) for CMR.

CMR performed well in LAV metric derivation on repeat processing of scans by the same operator (rho: 0.97) and by different operators (rho: 0.93) (Supplementary Table 4). In contrast, TTE performed worse (rho: 0.87 same operator; rho: 0.74 different operators).

None of the 17 studies reported on the scan-rescan repeatability nor on manufacturer reproducibility of the LAV measurement by either TTE, CMR or both.

### Quantification of LAV_max_

On average correlation was good between TTE and CMR values (Table 2, Supplementary Table 5).

**Table 2:** Left atrial quantification by TTE and CMR

Author, year	Indication (*n*)	Interval between TTE and CMR (days)^a^	TTE		CMR	
			Method	Value^a^	Method	Value^a^
LAV_max_reported (mL)						
Rahi W, 2024 [54]	Dyspnoea (RHC, PCWP > 15 mmHg) (33)	1 (3)	2DE biplane AL	96 ± 33	Biplane AL	141 ± 60
Rahi W, 2024 [54]	Dyspnoea (RHC, PCWP ≤ 15 mmHg) (46)	1 (3)	2DE biplane AL	66 ± 36	Biplane AL	81 ± 37
Agner BF, 2014 [55]	Atrial fibrillation (34)	7 ± 4	2DE biplane AL	60 ± 11	Multi-slice LA stack	73 ± 16
Rabbat MG, 2015 [56]	Atrial fibrillation (250 for CMR, 171 for TTE)	NR	2DE biplane AL	93 ± 27	Multi-slice LA stack	113 ± 37
Dell’Aquila AM, 2012 [60]	Post-heart transplant (35)	NR	2DE biplane Simpson’s	89 ± 38	Multi-slice LA stack	153 ± 70
Zhu S, 2020 [28]	Post-heart transplant (31)	1 (0)	2DE biplane AL	79 ± 22	Multi-slice LA stack	89 ± 23
Florescu DR, 2021 [27]	Non-disease specific (26)	1 (0)	2DE biplane Simpson’s	63 (53–78)	Multi-slice LA stack	76 (59–89)
Perez de Isla L, 2014 [30]	Non-disease specific (70)	< 21	2DE biplane AL	63 ± 27	Multi-slice LA stack	80 ± 29
Ramos JG, 2020 [44]	Non-disease specific (46)	3 (16)	2DE biplane AL	65 (52–84)	Biplane AL	80 (67–100)
Bias between LAV_max_ by TTE vs. by CMR (mL)						
Agner BF, 2014 [55]	Atrial fibrillation (34)	7 ± 4	2DE biplane AL	−14 ± 13	Multi-slice LA stack	–
Buechel RR, 2013 [29]	Atrial fibrillation (60)	1 (0)	2DE biplane Simpson’s	−13 ± 25	Multi-slice LA stack	
Rabbat MG, 2015 [56]	Atrial fibrillation (250 for CMR, 171 for TTE)	NR	2DE biplane AL	−24 ± 28	Multi-slice LA stack	
Dell’Aquila AM, 2012 [60]	Post-heart transplant (35)	NR	2DE biplane Simpson’s	−51 ± 31	Multi-slice LA stack	–
Zhu S, 2020 [28]	Post-heart transplant (31)	1 (0)	2DE biplane AL	−10 ± 16	Multi-slice LA stack	–
Florescu DR, 2021 [27]	Non-disease specific (26)	1 (0)	2DE biplane Simpson’s	−8 ± 9	Multi-slice LA stack	–
Perez de Isla L, 2014 [30]	Non-disease specific (70)	< 21	2DE biplane AL	−16 ± 26	Multi-slice LA stack	–
Mor-Avi V, 2012 [21]	Non-disease specific (92)	NR	2DE biplane AL	−31 ± 25	Multi-slice LA stack	–
LAVi reported (mL/m ^2^)						
Backhaus SJ, 2023 [33]	HFpEF (by RHC or stress TTE) (34)	NR	2DE biplane Simpson’s	44 (37–54)	Multi-slice LA stack	35 (30–43)
Backhaus SJ, 2023 [33]	Non-HFpEF Dyspnoea (34)	NR	2DE biplane Simpson’s	36 (29–41)	Multi-slice LA stack	28 (22–35)
Ng MY, 2023 [53]	HFpEF (by RHC) (53)	1 (0)	2DE biplane AL	52 (38–65)	Biplane AL	62 (48–86)
Ng MY, 2023 [53]	Non-HFpEF Dyspnoea (38)	1 (0)	2DE biplane AL	37 (31–42)	Biplane AL	44 (35–51)
Ng MY, 2023 [53]	Healthy (17)	1 (0)	2DE biplane AL	29 (24–37)	Biplane AL	43 (36–53)
Rahi W, 2024 [54]	Dyspnoea (RHC, PCWP > 15 mmHg) (33)	1 (3)	2DE biplane AL	47 ± 19	Biplane AL	71 ± 34
Rahi W, 2024 [54]	Dyspnoea (RHC, PCWP ≤15 mmHg) (46)	1 (3)	2DE biplane AL	32 ± 16	Biplane AL	43 ± 19
Buechel RR, 2013 [29]	Atrial fibrillation (60)	1 (0)	2DE biplane Simpson’s	51 ± 15	Multi-slice LA stack	58 ± 17
Chung, 2021 [57]	HCM, Sarcomere+ (67 for TTE, 42 for CMR)	NR	2DE ellipsoid	41 ± 22	Single plane AL	78 ± 44
Chung, 2021 [57]	HCM, Sarcomere- (145 for TTE, 93 for CMR)	NR	2DE ellipsoid	35 ± 15	Single plane AL	67 ± 23
Ricci F, 2019 [58]	HCM (Increased LAP, grade II-III) (23)	5 (7)	2DE biplane AL	68 (45)	Biplane AL	69 ± 13
Ricci F, 2019 [58]	HCM (Normal LAP, grade I) (46)	5 (7)	2DE biplane AL	47 (14)	Biplane AL	50 ± 12
Isaac M, 2024 [59]	Stroke (44)	NR	2DE biplane AL	29 ± 13	Biplane AL	35 ± 11
Kühl JT, 2012 [61]	Post-STEMI (54 for CMR, 48 for TTE)	1 (0)	2DE biplane AL	36 ± 11	Multi-slice LA stack	47 ± 10
Ramos JG, 2020 [44]	Non-disease specific (46)	3 (16)	2DE biplane AL	35 (29–44)	Biplane AL	41 (33–51)
Chung H, 2021 [57]	Age-, sex-matched controls (30 for TTE only)	NR	2DE ellipsoid	Mean: 20 (SD: 3)	Single plane AL	NR
Bias between LAVi by TTE vs. by CMR (mL/m ^2^)						
Fujikura K, 2024 [32]	HCM (87)	0 [0, 9]	2DE biplane AL	−13 (LoA − 7 to 32)	Multi-slice LA stack	–
Isaac M, 2024 [59]	Stroke (44)	NR	2DE biplane AL	−6	Biplane AL	–
Kühl JT, 2012 [61]	Post-STEMI (48)	1 (0)	2DE biplane AL	24%	Multi-slice LA stack	–
Ramos JG, 2020 [44]	Non-disease specific (46)	3 (16)	2DE biplane AL	−7 ± 8	Biplane AL	–
Misclassification (%) of LAVi > 34ml/m^2^ by TTE, compared to CMR						
Perez de Isla L, 2014 [30]	Non-disease specific (70)	< 21	2DE biplane AL	53 [95%CI 41, 64]	Multi-slice LA stack	–
Mor-Avi V, 2012 [21]	Non-disease specific (92)	NR	2DE biplane AL	29 [95%CI 20, 39]	Multi-slice LA stack	–
Fujikura K, 2024 [32]	HCM (87)	0 [0, 9]	2DE biplane AL	34 [95%CI 25, 45]	Multi-slice LA stack	–
Prognostic accuracy for hospitalisation for cardiovascular disease (Hazard ratio for LAV_max_)						
Backhaus SJ, 2023 [33]	Non-HFpEF Dyspnoea (34)	NR	2DE biplane Simpson’s	1.03 (95%CI 1.01–1.06)	Multi-slice LA stack	1.03 (95%CI 1.01, 1.06)
Prognostic accuracy for hospitalisation for cardiovascular disease (Hazard ratio for LAVi)						
Backhaus SJ, 2023 [33]	Non-HFpEF Dyspnoea (34)	NR	2DE biplane Simpson’s	1.06 (95%CI 1.01–1.11)	Multi-slice LA stack	1.06 (95%CI 1.01, 1.12)

^a^Data are shown as mean ± SD, median (interquartile range. For the mean bias the SD reported is the SD of mean differences

2DE: two-dimensional echocardiography, AL: area length method; LA: left atrial; NR: not reported. CI: confidence intervals

**Fig. 2:**
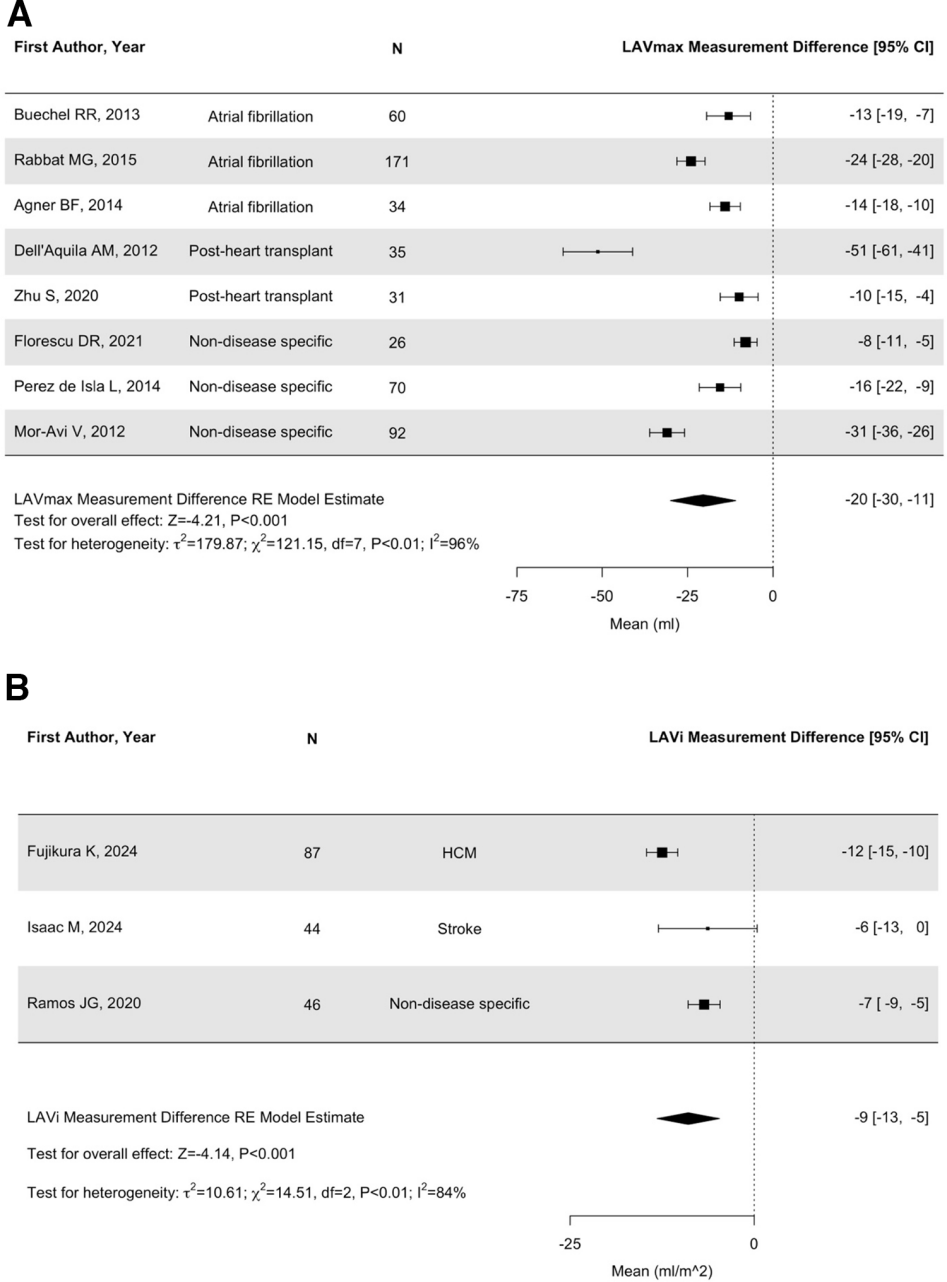

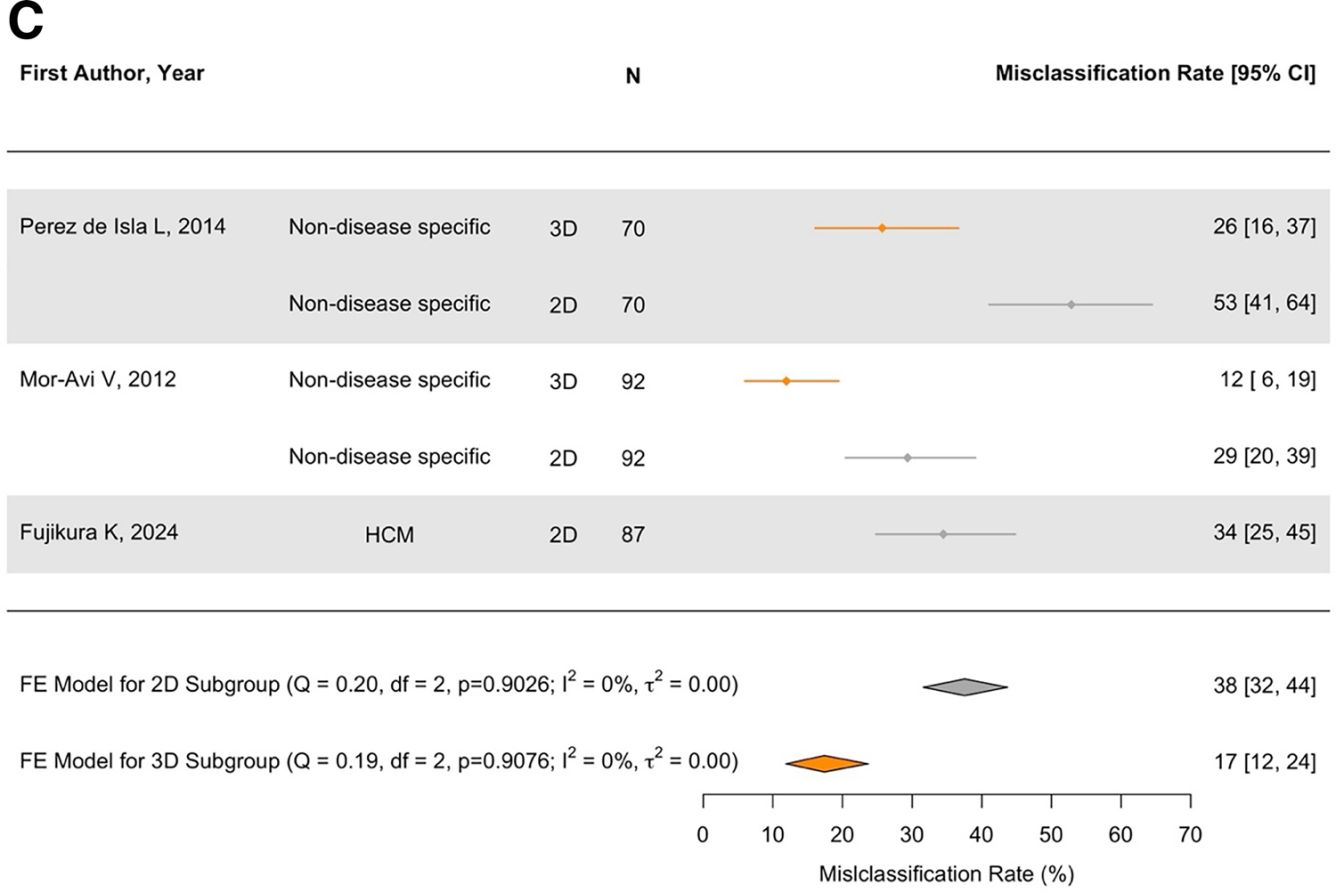
(**A**) Measurement difference between TTE and CMR for LAVmax. (**B**) Measurement difference between TTE and CMR for LAVi. (**C**) Misclassification of LA enlargement by TTE (grey for 2D and orange for 3D TTE). The rate of misclassification by 2DE was 38%. 3DE studies reported reduced misclassification rates by about 2-fold (17%)

In 8/8 studies reporting paired LAV_max_ values, those derived by TTE were lower than those by CMR. The LAV_max_ derived by TTE was 68mL [95%CI 53, 82] vs. CMR was 83mL [95%CI 64, 101]. On a patient-by-patient level, the TTE LAV_max_ values differed by −20 mL [95% CI −30, −10] (*p* < 0.001) from those by CMR (Fig. 2 A). In a supgroup analysis in individuals with AF, the TTE LAVmax values differed by −17mL [95% CI −24, −10] (*p* < 0.001) from LAVmax by CMR (Supplementary Fig. 2).

The underestimation of LAV_max_ by TTE occurred irrespective of CMR method (biplanar or multi-slice stack). The underestimation of LAV_max_ remained in 3/4 studies where 3D TTE was also used [27, 28] and [29]; (Supplementary Table 6).

### Quantification of LAVi

Nine studies reported paired LAVi by both CMR and TTE (Table 2, Supplementary Table 6). In 8/9 studies across all indications and subgroups, mean LAVi values reported by TTE (37mL/m^2^ [95%CI 30, 44]) were lower than by CMR (43mL/m^2^ [95%CI 36, 51]). On a patient-by-patient level, the TTE LAVi values differed by −9 mL/m^2^ [95% CI −13, −5] (*p* < 0.001) from those by CMR (Fig. 2B).

###  Classification of LA enlargement

Three studies reported that TTE misclassified patients according to the > 34 mL/m^2^ LAVi threshold for LA enlargement, referring to CMR LAVi as the gold standard [30–32] (Table 2). The rate of misclassification by TTE was 38% [95%CI 25, 52]. Two studies also reported on 3D TTE; the misclassification rates were still evident but reduced by about 2-fold (from 53 to 26% and from 29 to 12%, in [30] and [21], respectively) (Fig. 2C).

### Prognostic accuracy of LAV

Only a single study examined prognostic accuracy with LAV [33]. CMR and TTE predicted risk of CVD hospitalisation equally (LAVi by CMR HR 1.06 [95%CI 1.01, 1.12] and by TTE HR 1.06 [95%CI 1.01–1.11]) (Table 2).

## Discussion

This is the first systematic review of LAV measurement by TTE versus CMR within the same subjects, as performed in 17 studies comprising 1203 adults with different cardiac indications. Our analysis demonstrated 3 important findings. Firstly, TTE had a higher measurement failure rate compared to CMR (6% vs. 1%), mainly due to poor image quality and operator variability. Overall, consistently better inter-operator and intra-operator variability was seen by CMR. Secondly, LAV quantification by 2D TTE significantly underestimates LAV and LAVi values when compared to the gold standard CMR, regardless of CMR method used. Thirdly, 2D TTE misclassified 1 in every 3 patients.

### Differences in technical performance

This work demonstrated better technical performance with CMR, therefore reliance on TTE has implications both for clinical trials and healthcare. The reliability of echocardiography varies according to the density and depth of tissue through which the ultrasound (US) signal must pass. With the LA positioned distally to the US beam, image resolution will inevitably be compromised leading to the increased measurement failure rates seen using TTE. In a study of 2042 residents in Olmsted County Minnesota, 19% (365) patients did not have adequate measures of LAVi and diastolic dysfunction using TTE [34]. In clinical trials for HFpEF, this may also be compounded by obesity. In a study of over 1000 TTEs from 2022, 13% of scans were labelled as ‘non-diagnostic’ in people with obesity, compared to only 8% in people without obesity [35]. Nevertheless, MR image quality may also be compromised in patients with arrhythmias such as AF [36]. AF poses challenges for CMR acquisition due to irregular R-R intervals, which can compromise image quality through motion artifacts and affect the accuracy of volumetric measurements. However, in this review, of the 3 studies on AF, all images were of sufficient quality for analysis except for only 2 cases.

CMR has demonstrated better technical success rates across operators or by the same operator, compared to TTE. We found that 3D TTE underestimated LAV less than 2D. This is not surprising as unlike 2DE, 3D echocardiography does not depend on geometric assumption, thus, it is more accurate and reproducible than 2DE [20]. While potentially offering improved volumetric assessment compared to 2D methods, it remains highly dependent on image quality. Both TTE methods require good spatial and temporal resolution of the image and, unlike CMR, a standardised method for 3D post processing analysis of LAV is not yet adopted [20]. Among the CMR methods, the multi-slice LA stack method is regarded as most reliable [37] because it tracks endocardial borders of successive slices across the whole atrium, overcoming the geometric assumptions of the biplane methods [37].

### Diagnosis of LA enlargement

Across cardiac diseases, TTE was inferior to CMR in this work, supporting CMR as the gold standard technique for LAV measurement. We found that TTE underestimated LAV and LAVi in almost all studies, resulting in missed diagnoses of LA enlargement in 38% of patients by 2D TTE that were still missed by 3D TTE in half. The systematic underestimation of LA volumes observed in the present meta-analysis is likely contributed to by foreshortening of the left atrium obtained from the standard apical four- and two-chamber TTE views. Proper LA-focused views are achieved by adjusting TTE transducer until the maximum LA long axis is identified. The use of atrial-focused views has been shown to provide larger volumes than the standard apical views and are in better agreement with 3DE [38]. Although this technique could potentially reduce measurement disparity between TTE and CMR, it still relies on good TTE image quality.

A relevant application is in HFpEF, where LA enlargement is a diagnostic criterion and the scale of clinical trials requires optimal technical performance. Diagnosis of LA enlargement in current guidelines for HFpEF is based on the observed association of LAVi above 34 ml/m^2^ measured by TTE with cardiovascular events and mortality [39–41]. With the current review, the threshold for stratification of LA enlargement may be better defined by CMR.

In HFpEF the underlying impaired cardiac relaxation results in heterogeneous clinical presentation with multiple features of LV dysfunction and/or increased LV filling pressure. Current recommendation advocates for meticulous pathophysiological-based phenotyping to enhance patient characterization and facilitate the individualization of treatment [3]. Cardiac dysfunction in HFpEF is multi-layered and identified by some combination of cardiac stiffness, reduced filling and emptying in the LV, increased pressure in ventricle and atrium, hypertrophy and remodelling on the LV and atrium eventually affecting the right ventricle and atrium. The multiparametric approach of diastolic function is based on the assessment of LA volume index, maximal tricuspid regurgitation velocity, E/e’ ratio and mitral annular velocity (e’) by TTE [42]. Nevertheless, CMR measurements of diastolic function have been shown to be comparable with echo, thus CMR-derived diastolic indices may be integrated in future practice [43, 44]. Echo-based diastolic function assessment is dependent on good image quality, and this is often impaired in obesity which is highly prevalent in HFpEF.

Beyond LA enlargement, CMR is the gold standard for evaluation of known early features such as cardiac stiffness (abnormal global longitudinal strain (GLS), and cardiac hypertrophy and elevated LV mass [45]. This is particularly relevant because obesity and diabetes are clinical comorbidities in 40–50% of people with HFpEF and in those patients due to their body habitus the accurate assessment of LAV may be possible by CMR. Additionally, both inflammation [46] and epicardial fat deposition contribute to localized dysfunction, measured by CMR in a recent HFpEF clinical trial with tirzepatide [45], so CMR could further improve the diagnostic algorithm beyond LA enlargement. CMR techniques may be unsuitable for routine application in large population due to high cost and limited availability. However, as CMR is more accurate and reproducible, it is more cost-effective given substantial reduction in sample sizes required in research studies compared to 2DE [47]. In clinical settings, CMR is preferred when small changes are of clinical importance for making treatment decision [48]. Other recognised limitations of CMR are patient-specific barriers, including claustrophobia, implanted devices, or inability to lie flat for extended periods, also restrict partially its feasibility.

### Limitations

While the literature search was comprehensive, there may be studies not found using our search criteria, although experts in the field were consulted to add to the studies identified. There was a paucity of articles solely on HFpEF and the studies we found were cross-sectional and on 2D TTE. The effect of differences in hardware or variability in image acquisition (scan-rescan repeatability) on LAV quantification was not reported in any study, despite their importance for large-scale applications as recommended by the Quantitative Imaging Biomarkers Alliance [49]. However, test-retest repeatability has been reported in independent studies of one technology in the absence of a head-to-head comparison; for CMR biplane method LAVmax value ICC 0.89 [50] and for 2D TTE LAVmax ICC 0.92 [51]. Only 1 study reported on association to cardiovascular outcomes using both TTE and CMR and only 1 study compared CMR and TTE in healthy controls. While independent studies have found associations of LAVi to outcomes by TTE [39–41], equivalent studies are needed to identify optimal CMR prognostic biomarkers for HFpEF and the clinical impact of observed LAVi underestimation by TTE, given the improved technical performance of CMR reported here. Only 10/17 studies reported on BMI or BSA and 0/17 studies reported on ethnicity despite their influence on severity and course of cardiometabolic disease. These limitations should be addressed to inform HFpEF clinical trials with prospective research on technical performance, including on 3D TTE, and change over time. We did not specifically look at cardiac computed tomography angiography (CCTA) as it is beyond the scope of this review. CCTA offers true 3D isotropic spatial resolution for LA evaluation, superior to echocardiography and standard CMR protocols. It can be performed faster than echo and CMR, with sufficient temporal and spatial resolution [52]. Cardiac CT is however limited by exposure to radiation making it less suitable for repeated studies.

### Conclusion

In this meta-analysis of paired studies, CMR has superior technical performance over TTE for diagnosis of LA enlargement and TTE misdiagnosed LA enlargement in 38% of cases. LA enlargement is a measure of cardiac dysfunction and contributes to pathophysiology in obesity and HFpEF. The limitations of TTE should be considered as we design clinical pathways for HFpEF, and interventional clinical trials.

## Electronic supplementary material

Below is the link to the electronic supplementary material.

Supplementary Material 1

## Data Availability

No datasets were generated or analysed during the current study.

## Abbreviations

AF: Atrial fibrillation
AL: Area length
BMI: Body mass index
BSA: Body surface area
CI: Confidence interval
CMR: Cardiovascular magnetic resonance
EF: Ejection fraction
HCM: Hypertrophic cardiomyopathy
HF: Heart failure
HFpEF: Heart failure preserved ejection fraction
IQR: Inter-quartile range
LA: Left atrium
LAP: Left atrial pressure
LAV: Left atrial volume
LAVi: Left atrial volume index
LoA: Limits of agreement
NR: Nsot reported
PCWP: Pulmonary capillary wedge pressure
RHC: Right heart catheterization
PICOS: Population, intervention, comparison, outcome and study type
PRISMA: Preferred Reporting Items for Systematic Reviews and Meta-Analyses
SD: Standard deviation
STEMI: ST segment elevation myocardial infarction
TTE: Transthoracic echocardiography
