# Supplementary material for: Left atrial volume quantification by transthoracic echocardiography versus cardiovascular magnetic resonance: a systematic review and meta-analysis
Source: Int J Cardiovasc Imaging. 2025 Jul 23;41(9):1657–69. doi: 10.1007/s10554-025-03455-1 (PMC12405336; doi:10.1007/s10554-025-03455-1)
Supplement: Supplementary file 1 — Supplementary Material 1 [file 10554_2025_3455_MOESM1_ESM.docx]

**Left atrial volume quantification by transthoracic echocardiography versus cardiovascular magnetic resonance: A systemic review and meta-analysis**

|  | **Legend** | **Pages** |
| --- | --- | --- |
| Supplementary Figure 1 | PRISMA flow diagram on study selection | 2 |
| Supplementary Figure 2 | Measurement difference between TTE and CMR for LAVmax in studies of individuals with AF | 3 |
| Supplementary Table 1 | Detailed characteristics of selected studies | 4 |
| Supplementary Table 2 | Quality assessment of selected studies, showing answers to specific domain questions and corresponding overall scores of study quality. | 7 |
| Supplementary Table 3 | Failure rate of left atrial quantification by TTE and CMR | 10 |
| Supplementary Table 4 | Inter- and intra-operator variability of LAV measurement by CMR and by TTE | 11 |
| Supplementary Table 5 | Correlation between TTE and CMR LAV values | 13 |
| Supplementary Table 6 | Left atrial quantification by TTE and CMR from selected studies, with additional subsets | 14 |
| Supplementary References |  | 17 |

**Supplementary Figure 1.** PRISMA flow diagram on study selection

**Supplementary Figure 2.** Measurement difference between TTE and CMR for LAVmax in studies of individuals with AF

**
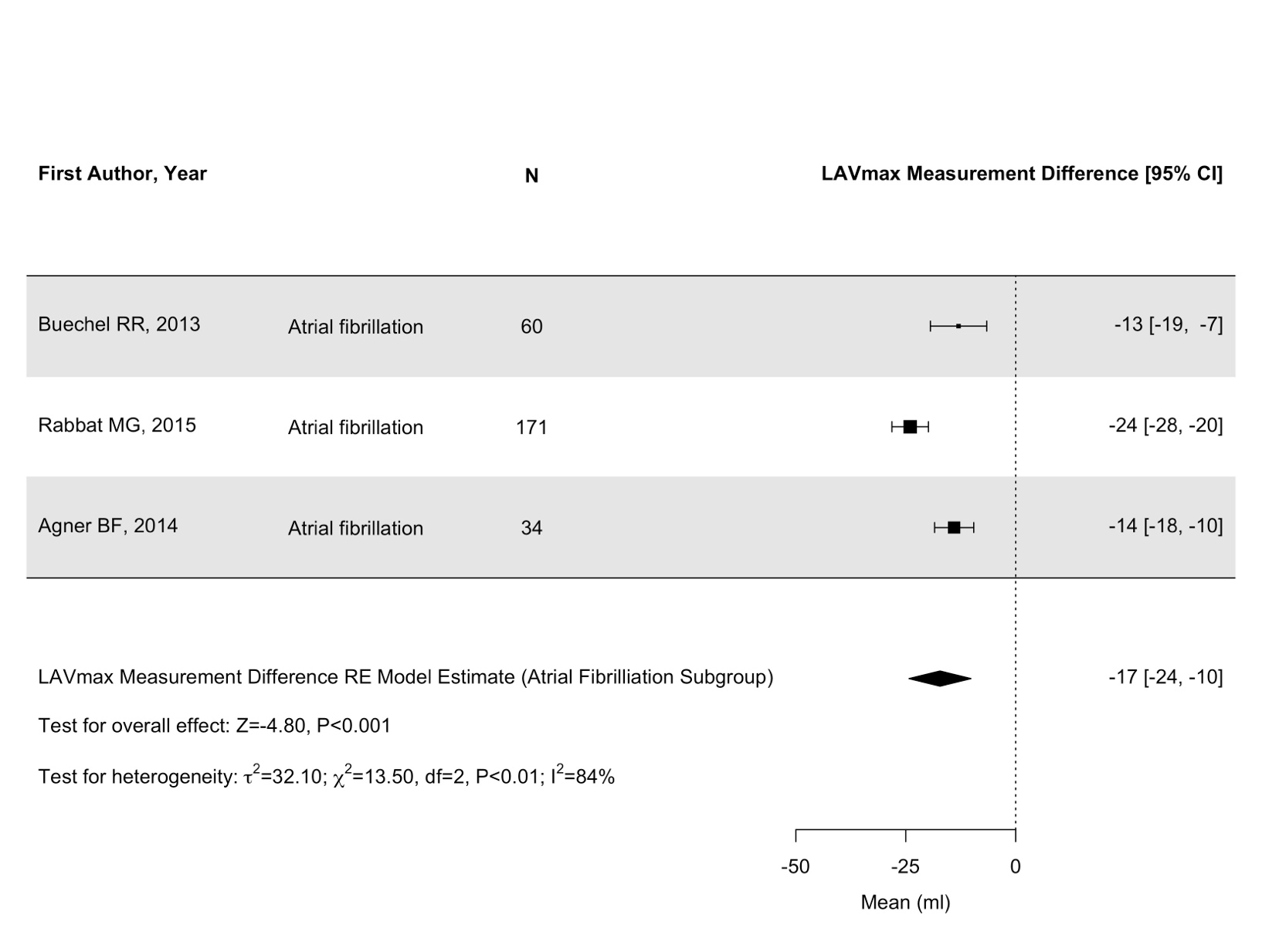
**

**Supplementary Table 1:** Detailed characteristics of selected studies

| **Author** | **Year, country** | **Study design** | **Indication** | **Sample size, n** | **Population characteristics** |
| --- | --- | --- | --- | --- | --- |
| **Dyspnoea and suspected HFpEF** | | | | | |
| Ng MY et al. (1) | 2023, Hong Kong | Prospective, case-control study | Patients suspected to have HFpEF, underwent echo and CMR within 24 hours | 108 (53 HFpEF, 38 non-HFpEF, 17 normal) | HFpEF (age 78 years, female 60%, BMI 20.7 kg/m^2^)  Non-HFpEF (age 70 years, female 55%, BMI 27.4 kg/m^2^)  Normal (age 64 years, female 65%, BMI 20.7 kg/m^2^) |
| Backhaus SJ et al. (2) | 2023, Germany | Prospective, case-control study with clinical outcome follow up | Patients with HFpEF and non-cardiac dyspnoea underwent echo, RHC and CMR | 68 (34 HFpEF, 34 non-cardiac dyspnoea) | HFpEF (age 69 years, male/female 36%, BMI 28.7 kg/m^2^)  Non-cardiac dyspnoea (age 66 years), male/female 79, BMI 27.6 kg/m^2^) |
| Rahi W et al. (3) | 2024, USA | Retrospective single centre study | Patients with dyspnoea and LVEF >50% underwent RHC, CMR and echo within one week | 79 | Mean age 54.6 years, female 58%, BMI 28.6 kg/m^2^ |
| **Atrial Fibrillation** | | | | | |
| Agner BF et al. (4) | 2014, Denmark | Prospective study | Patients with permanent AF underwent echo, CMR and MDCT withing 7 days | 34 | Mean age 65 years, male 22%, BSA 2.0 m^2^ |
| Buechel RR et al. (5) | 2013, Switzerland | Prospective study | Patients with AF scheduled for PV isolation underwent 2/3D echo and CMR within the same day | 60 | Mean age 61 years, male 70%, BMI 27 kg/m^2^ |
| Rabbat MG et al. (6) | 2015, USA | Prospective, single centre | Patients with AF underwent CMR and echo prior to ablation procedure | 250 | Mean 58 years old, male 72%, BMI 29 kg/m^2^ |
| **Cardiomyopathy** | | | | | |
| Chung H et al, (7) | 2021, Korea | Single centre, observational HCM registry | HCM Registry, with genetic testing | HCM gene+ (67 TTE, 42 CMR). HCM gene- (145 TTE, 93 CMR). Controls 30 (TTE only) | HCM gene+: mean age 55 ± 14 years, female 37%  HCM gene-, mean age 61 ± 13 years, female 26%  Controls, mean age 60 ± 3 years |
| Ricci F et al. (8) | 2019, United Kingdom | Retrospective | HCM outpatients underwent echo and CMR | 69 | Mean age 58 years, male 83%, BMI 29 kg/m^2^ |
| Fujikura K et al. (9) | 2024, USA | Prospective, case-control study | Patients with suspected or diagnosed cardiomyopathy, underwent CMR and echo on the same day | 87 (cardiomyopathy 63, healthy volunteers 24) | Mean age 45 years, male 56.8%, BSA 1.9 m^2^ |
| **Stroke** | | | | | |
| Isaac M et al. (10) | 2024, USA | Retrospective study | Patients with stroke or stroke-like symptoms | 44 | Mean age 60 years, female 41%, BSA 2.07 m^2^ |
| **Post STEMI** | | | | | |
| Kühl JT et al. (11) | 2012, Denmark | Prospective | Post STEMI 3 months. MSCT, TTE, CMR performed within 9 days. TTE and CMR on the same day | 54 | Mean age 61 years, female 24%, BSA 2.1 m^2^, BMI 28 kg/m^2^ |
| **Post heart transplant** | | | | | |
| Dell'Aquila AM et al. (12) | 2012, Spain | Retrospective study | Patients post orthotopic heart transplant (standard vs bicaval technique) | 35 eligible for imaging study (16 standard group, 19 bicaval group) | Standard group (age 47.7 years, male 94%)  Bicaval group (age 55.4 years, male 95%) |
| Zhu S et al. (13) | 2020, China | Prospective study | Heart transplant recipients, underwent 2/3D echo and CMR on the same day | 31 | Mean age 45.5 years, male 74%, BSA 1.67 m^2^ |
| **Non-disease specific** | | | | | |
| Mor-Avi V et al. (14) | 2012, USA, Australia, Germany, Austria | Prospective, multicentre study | Patients with a wide range of LAV who underwent CMR and echo on the same day | 92 | Mean age 48 years, female 38%, BSA 1.72 m^2^ |
| Perez de Isla L et al. (15) | 2014, Spain | Prospective, observational, single centre study | Consecutive patients referred for CMR. CMR and echo study performed within 3 weeks. | 70 | Mean age 56 years, male 60%, BSA 1.74 m^2^ |
| Ramos JG et al. (16) | 2020, Sweden | Prospective, observational study | Clinically referred patients for CMR, and already had echo | 46 | Mean age 59 years, female 33%, BMI 26 kg/m^2^ |
| Florescu DR et al. (17) | 2021, Romania, Italy | Prospective study | Consecutive patients referred for clinically indicated echo and CMR within the same day | 198 had TTE, 26 paired for CMR | 198/210 patients, age 67 years, male 64%, BSA 1.83 m^2^ |

**Supplementary Table 2**: Quality assessment of selected studies, showing answers to specific domain questions and corresponding overall scores of study quality.

| **Author, year** | **Selection** | | | | **Comparability** | **Exposure/ Outcomes** | | | **Study Score^17^** |
| --- | --- | --- | --- | --- | --- | --- | --- | --- | --- |
| Case-control studies | | | | | | | | |  |
|  | Case definition^1^ | Case representation^2^ | Control selection^3^ | Control definition^4^ | Cases vs controls for the design intended in the meta-analysis^5^ | Overall ascertainement^6^ | Cases vs controls for ascertainement^7^ | Non-response rate^8^ |  |
| Backhaus SJ, 2023 (2) | a | a | a | a | c | a | a | a | 10 |
| Chung, 2021 (7) | b | b | a | a | a | e | b | c | 3 |
| Ng MY, 2023 (1) | a | a | a | a | a | a | a | a | 8 |
| Rahi W, 2024 (3) | a | a | a | a | a | a | a | a | 8 |
| Ricci F, 2019 (8) | a | a | a | a | a | a | a | c | 7 |
| Cohort studies | | | | | | | | |  |
|  | Exposed cohort definition^9^ | Non-exposed cohort definition^10^ | Ascertainment of LAV acquisition by CMR or TTE^11^ | LAV values by CMR & TTE at start of study^12^ | Exposed vs non-exposed for the design intended in the meta-analysis^13^ | Ascertainment of LAV values^14^ | Follow up adequacy for ascertainment by both CMR and TTE^15^ | Follow up adequacy for both CMR and TTE in all subjects^16^ |  |
| Mor-Avi V, 2012 (18) | a | a | a | a | c | a | b | d | 8 |
| Perez de Isla L, 2014 (15) | a | a | a | a | a | b | b | d | 7 |
| Ramos JG, 2020 (16) | a | a | a | a | a | b | a | c | 9 |
| Zhu S, 2020 (13) | c | a | a | a | c | b | a | a | 10 |
| Kühl JT, 2012 (11) | a | a | a | a | c | a | a | a | 9 |
| Isaac M, 2024 (10) | b | a | a | a | a | a | b | d | 7 |
| Fujikura K, 2024 (9) | a | a | a | a | c | b | a | c | 10 |
| Agner BF, 2014 (4) | a | a | a | a | c | a | a | c | 10 |
| Buechel RR, 2013 (5) | a | a | a | a | a | a | a | a | 8 |
| Rabbat MG, 2015 (6) | a | b | a | a | a | b | b | d | 7 |
| Florescu DR, 2021 (17) | a | b | a | a | c | a | a | a | 9 |
| Dell'Aquila AM, 2012 (12) | b | a | a | a | a | a | b | d | 7 |

1. Adequacy of case definition: a) yes, with independent validation (e.g. >1 person/record/time/process to extract information, or reference to primary record source such as tests or medical/hospital records), b) yes, e.g. record linkage (e.g. ICD codes in database) or based on self reports; c) no description.
2. Adequacy of case representation: a) consecutive or obviously representative series of cases (all eligible cases with outcome of interest over a defined period of time, all cases in a defined catchment area, all cases in a defined hospital or clinic, group of hospitals, health maintenance organisation, or an appropriate sample of those cases, a random sample) or b) potential for selection biases or not stated.
3. Adequacy of control selection: a) community controls (i.e. same community as cases and would be cases if had outcome), b) hospital controls (within same community as cases (i.e. not another city) but derived from a separate or not fully overlapping population), c) no description.
4. Adequacy of control definition: a) no history of disease relevant to case definition or b) history not stated.
5. Comparability of cases and controls by matching and/or confounders adjustment: a) they were matched for LAV measurement (including bias), b) matched for failure rate, c) both a & b were met, d) cases and controls were not matched for either LAV nor failure rate.
6. Overall ascertainment of LAV measurements: a) from secure record, CMR and TTE within 7 days on average, or b) not within 7 days.
7. Ascertainment of LAV measurements by TTE vs CMR: a) yes ascertained similarly for cases and controls or b) no, not similarly.
8. Non-response rate (whether sample size was the same for CMR and TTE): a) same for both groups, b) different but demographics/measurements described and c) different but no designation.
9. Adequacy of cohort exposed to CMR: a) truly representative of average cardiology patients in the community, b) somewhat representative of the average cardiology patients, c) a selected subset or d) no description provided.
10. Adequacy of non-exposed cohort (exposed to TTE): a) drawn from the same community as those exposed to CMR or unpaired in over 30% of participants, b) drawn form a different source, c) a selected subset or d) no description provided.
11. Ascertainment of LAV acquisition by CMR or TTE: a) with adequate methodology from secure records, b) poor methodology, c) no description.
12. Study investigators with pre-existing knowledge of LAV values by CMR & TTE at the start of the study: a) no knowledge and b) knowledge, introducing bias.
13. Comparability of exposed (CMR) and non-exposed (TTE) cohorts by matching and/or confounders adjustment: a) matched for LAV measurement (including bias), b) matched for failure rate, c) both a & b were met, d) cases and controls were not matched for either LAV nor failure rate.
14. Overall ascertainment of LAV measurements: a) independent blinded assessment, b) record linkage or c) no description.]
15. Duration of follow-up period for ascertainment by both CMR and TTE: a) within 7 days on average or b) more than 7 days on average.
16. Duration of follow-up period for both CMR and TTE in all subjects: a) complete follow up and all subjects accounted for, b) subjects lost to follow up unlikely to introduce bias because only <10 % of subjects lost, c) follow up rate < 90% of subjects and no description of those lost or d) no description.
17. Studies with total scores ≥7/10 were considered good quality with low risk of bias.

**Supplementary Table 3:** Failure rate of left atrial quantification by TTE and CMR

| **Author, year** | **Indication** | **TTE** | | | **CMR** | | |
| --- | --- | --- | --- | --- | --- | --- | --- |
|  |  | **Method** | **Failures (rate as %)** | **Cause(s)** | **Method** | **Failures (rate as %)** | **Cause(s)** |
| Agner BF, 2014 (4) | Atrial fibrillation | 2DE biplane AL | 0/34 (0%) | - | Multi-slice LA stack | 6/40 (15%) | Claustrophobia, poor image quality |
| Kühl JT, 2012 (11) | Post-STEMI | 2DE biplane AL | 5/48 (10%) | Poor acoustic window | Multi-slice LA stack | 0/54 (0%) | - |
| Zhu S, 2020 (13) | Post-heart transplant | 2DE biplane AL | 4/35 (11%) | Poor image quality | Multi-slice LA stack | 2/37 (5%) | Inability for breath-hold |
| Florescu DR, 2021 (17) | Non-disease specific | 2DE biplane Simpson’s | 8/210 (4%) | Poor acoustic window, poor image quality, inadequate tracking | Multi-slice LA stack | 0/26 (0%) | - |
| Mor-Avi V, 2012 (18) | Non-disease specific | 2DE biplane AL | 15/107 (14%) | Poor image quality | Multi-slice LA stack | 0/92 (0%) | - |
| Fujikura K, 2024 (9) | HCM | 2DE biplane AL | 3/103 (3%) | Diastolic parameters not acquired | Multi-slice LA stack | 3/103 (3%) | Scanner software crashed, no paired TTE |
| Chung H, 2021 (7) | HCM | 2DE ellipsoid | NR | NR | Single-plane AL | 0/135 (0%) | - |

**Supplementary Table 4**: Inter- and intra-operator variability of LAV measurement by CMR and by TTE

| **Study** | **CMR** | **TTE (2D)** | **TTE (3D)** |
| --- | --- | --- | --- |
| **Intra-observer variability** | | |  |
| Agner BF et al. (4) | 0.96 | 0.61 |  |
| Buechel RR et al. (5) | 0.96 | - |  |
| Florescu DR et al. (17) | - | 0.99 | 0.995 |
| Rabbat MG et al. (6) | 0.99 (95%CI: 0.97-0.99) | 0.99 (95%CI: 0.96-0.99) |  |
| Perez de Isla L et al. (15) | - | 0.91 (95% CI: 0.76-0.97) | 0.99 (95% CI: 0.96-0.99) |
| Zhu S et al. (13) |  | 0.96 (95% CI: 0.93-0.98) | 0.98 (95% CI: 0.96-0.99) |
| **Inter-observer variability** | | |  |
| Agner BF et al. (4) | 0.93 | 0.52 |  |
| Buechel RR et al. (5) | 0.88 |  |  |
| Florescu DR et al. (17) | - | 0.988 | 0.995 |
| Rabbat MG et al. (6) | 0.96 (95%CI: 0.80-0.99) | 0.98 (95%CI: 0.82-0.99) |  |
| Ramos JG et al. (16) | 0.95 (95%CI: 0.82-0.98) |  |  |
| Perez de Isla L et al. (15) | - | 0.91 (95% CI: 0.73-0.97) | 0.99 (95% CI: 0.99-0.99) |
| Ng MY et al. (1) |  | 0.99 (95%CI: 0.98-0.99) |  |
| Zhu S et al. (13) |  | 0.93 (95% CI: 0.79-0.97) | 0.96 (95% CI: 0.91-0.99) |

**Supplementary Table 5**: Correlation between TTE and CMR LAV values

| **Author, year** | **Indication (n)** | **Methods** | **Correlation rho** |
| --- | --- | --- | --- |
| Fujikura K, 2024 (9) | Hypertrophic cardiomyopathy (87) | Biplane for TTE and CMR | 0.61 |
| Agner BF, 2014 (4) | Atrial fibrillation (34) | Biplane TTE; Multi-slice CMR | 0.59 |
| Buechel RR, 2013 (5) | Atrial fibrillation (60) | Biplane TTE; Multi-slice CMR | 0.70 |
| Rabbat MG, 2015 (6) | Atrial fibrillation (250 for CMR, 171 for TTE) | Biplane TTE; Multi-slice CMR | 0.66 |
| Kühl JT, 2012 (11) | Post-STEMI (54 for CMR, 48 for TTE) | Biplane TTE; Multi-slice CMR | 0.71 |
| Zhu S, 2020 (13) | Post-heart transplant (31) | Biplane TTE; Multi-slice CMR | 0.76 |
| Dell'Aquila AM, 2012 (12) | Post-heart transplant (35) | Biplane TTE; Multi-slice CMR | 0.57 |
| Mor-Avi V, 2012 (14) | Non-disease specific (92) | Biplane TTE; Multi-slice CMR | 0.74 |
| Perez de Isla L, 2014 (15) | Non-disease specific (70) | Biplane TTE; Multi-slice CMR | 0.54 |
| Ng MY, 2023 (1) | Healthy (17) | Biplane for TTE and CMR | 0.86 |

**Supplementary Table 6**: Left atrial quantification by TTE and CMR from selected studies, with additional subsets

| **Author, year** | **Time between echo and CMR (days)** | **Echocardiography** | | | **CMR** | | |
| --- | --- | --- | --- | --- | --- | --- | --- |
|  |  | **Vendor** | **Analysis method** | **LAV values** | **Vendor** | **Analysis method** | **LAV values** |
| Ng MY, 2023 (1) | Median 1 (IQR 0) | GE | 2DE biplane AL | 1. HFpEF LAVi 51.6 mL/m2 2. Non-HFpEF LAVi 37.2 mL/m2 3. Normal volunteers LAVi 29.3 ml/m2 | Philips 3T and  GE 3T | Biplane AL | 1. HFpEF LAVi 61.9 mL/m2 2. Non-HFpEF LAVi 43.7 mL/m2 3. Normal volunteers LAVi 42.6 mL/m2 |
| Backhaus SJ, 2023 (2) | NR | NA | 2DE biplane Simpson’s | 1. HFpEF LAVi 43.8 mL/m2 2. Non-cardiac dyspnoea LAVi 36.2 mL/m2 | Siemens 3.0T | Multi-slice LA stack | 1. HFpEF LAVi 35.3 mL/m2 2. Non-cardiac dyspnoea LAVi 28.2 mL/m2 |
| Rahi W, 2024 (3) | Median 1 (IQR 3) | NA | 2DE biplane AL | 1. LAVmax 75.7 ± 6.7 ml 2. LAVi 38.3 ± 18.8 ml/m2 | Siemens 1.5T and 3.0T | Biplane AL | 1. Biplane LAVmax 106.2 ± 56 ml 2. Biplane LAVi 54.5 ± 29.6 ml/m2 |
| Agner BF, 2014 (4) | Mean 7 (SD 4) | Philips | 2DE biplane AL | LAVi max 60 ± 11 ml/m2,  LAVi min 50 ± 12 ml/m2 | Siemens 1.5T and 3.0T | Multi-slice LA stack LAVi | LAVi max 73 ± 16 ml/m2,  LAVi min 64 ± 15 ml/m2 |
| Buechel RR, 2013 (5) | Median 1 (IQR 0) | Philips | 1. 2DE biplane AL 2. 2DE biplane Simpson’s 3. 3DE | 1. 2DE biplane LAVi 51 mL/m2, 2. 2DE Simpson's LAVi 51 mL/m2 3. PE (prolate ellipse) LAVi 31 ml/m2 4. 3DE (4D analysis) LAVi 56 ml/m2 5. 3DE (QLAB analysis) LAVi 49 mL/m2 | Siemens 1.5T | Multi-slice LA stack | LAVi 58 mL/m2 |
| Rabbat MG, 2015 (6) | NR | NA | 2DE biplane AL | LAV 93.1 ± 27.4 mL | Siemens 3.0T | 1. Multi-slice LA stack 2. Biplane AL | 1. LAV 112.7 ± 36.7 mL 2. LAV 126.1 ± 44.1 mL |
| Chung H, 2021 (7) | NR | NA | 2DE ellipsoid | Mean: 20 (SD: 3) mL/m2 | Siemens 1.5T | Single plane AL | NR |
| Ricci F, 2019 (8) | Median 5 (IQR 7) | GE Vivid E9 | 2DE biplane AL | LAVi 49 ml/m2 | Siemens 1.5T | Biplane AL | LAVi 56 ± 12 ml/m2 |
| Isaac M, 2024 (10) | NR | NA | 2DE biplane AL | LAVi 28.81ml/m2 | NA | Biplane AL | LAVi 34.97ml/m2 |
| Kühl JT, 2012 (11) | Median 1 (IQR 0) | Philips | 2DE biplane AL | 1. LAV max 74ml 2. LAVi 36 ml/m2 | Siemens 1.5T | Multi-slice LA stack | 1. LAV max 98 ml 2. LAVi 47 ml/m2 |
| Dell'Aquila AM, 2012 (12) | NR | Philips | 2DE biplane Simpson’s | LAVmax 88 ± 38 mL, LAVmin 71 mL | Siemens 1.5T | Multi-slice LA stack | LAVmax 153 ± 69 mL, LAVmin 119 mL |
| Zhu S, 2020 (13) | Median 1 (IQR 0) | Philips | 1. 2DE biplane AL 2. 3DE | 1. LAV 79.1 ± 21.9 ml 2. LAV 3DE 82.6 ± 22 ml | Siemens 1.5T | Multi-slice LA stack | LAV 88.9 ± 23.0 mL |
| Fujikura K, 2024 (9) | 0 [0, 9] | Philips | 2DE biplane AL | NR | Siemens 3.0T | Multi-slice LA stack | NR |
| Mor-Avi V, 2012 (14) | NR | Philips | 2DE biplane AL | -31 ±25 mL | GE, Philips and Siemens 1.5T | Multi-slice LA stack | NR |
| Perez de Isla L, 2014 (15) | < 21 | Toshiba | 1. 2DE biplane AL 2. 3DE | 1. LAV 63.33 ± 26.82 mL 2. LAV 79.8 ± 29 mL | GE 1.5T | Multi-slice LA stack | LAVmax 79.8 ± 28.99 mL |
| Ramos JG, 2020 (16) | Median 3 (IQR 16) | Philips | 2DE biplane AL | 1. LAV 65 (52-84) mL 2. LAVi 35 (29-44) ml/m2 | Siemens 1.5T and 3.0T | Biplane AL | 1. LAV 80 (67-100) mL 2. LAVi 41 (33-51) ml/m2 |
| Florescu DR, 2021 (17) | Median 1 (IQR 0) | Toshiba | 1. 2DE biplane Simpson’s 2. 3DE | 1. LAV 63 mL IQR (53-78) 2. LAV 72 mL IQR (57-87) | Siemens 1.5T | Multi-slice LA stack | LAV 76 mL IQR (59-89) |

**Supplementary References**

1. Ng MY, Kwan CT, Yap PM et al. Diagnostic accuracy of cardiovascular magnetic resonance strain analysis and atrial size to identify heart failure with preserved ejection fraction. Eur Heart J Open 2023;3:oead021.

2. Backhaus SJ, Lange T, Schulz A et al. Cardiovascular magnetic resonance rest and exercise-stress left atrioventricular coupling index to detect diastolic dysfunction. Am J Physiol Heart Circ Physiol 2023;324:H686-h695.

3. Rahi W, Hussain I, Quinones MA, Zoghbi WA, Shah DJ, Nagueh SF. Noninvasive Prediction of Pulmonary Capillary Wedge Pressure in Patients With Normal Left Ventricular Ejection Fraction: Comparison of Cardiac Magnetic Resonance With Comprehensive Echocardiography. J Am Soc Echocardiogr 2024;37:486-494.

4. Agner BF, Kühl JT, Linde JJ et al. Assessment of left atrial volume and function in patients with permanent atrial fibrillation: comparison of cardiac magnetic resonance imaging, 320-slice multi-detector computed tomography, and transthoracic echocardiography. Eur Heart J Cardiovasc Imaging 2014;15:532-40.

5. Buechel RR, Stephan FP, Sommer G, Bremerich J, Zellweger MJ, Kaufmann BA. Head-to-Head Comparison of Two-Dimensional and Three-Dimensional Echocardiographic Methods for Left Atrial Chamber Quantification with Magnetic Resonance Imaging. Journal of the American Society of Echocardiography 2013;26:428-435.

6. Rabbat MG, Wilber D, Thomas K et al. Left atrial volume assessment in atrial fibrillation using multimodality imaging: a comparison of echocardiography, invasive three-dimensional CARTO and cardiac magnetic resonance imaging. Int J Cardiovasc Imaging 2015;31:1011-8.

7. Chung H, Kim Y, Park CH et al. Contribution of sarcomere gene mutations to left atrial function in patients with hypertrophic cardiomyopathy. Cardiovasc Ultrasound 2021;19:4.

8. Ricci F, Aung N, Thomson R et al. Pulmonary blood volume index as a quantitative biomarker of haemodynamic congestion in hypertrophic cardiomyopathy. Eur Heart J Cardiovasc Imaging 2019;20:1368-1376.

9. Fujikura K, Sathya B, Acharya T et al. CMR provides comparable measurements of diastolic function as echocardiography. Sci Rep 2024;14:11658.

10. Isaac M, Kumar SA, Petroski GF, Shinn A, Mehra A, Gomez CR. Assessing left atrial size and pump function in ischemic stroke patients: Is cardiac MRI superior to transthoracic echocardiography? J Stroke Cerebrovasc Dis 2024;33:107674.

11. Kühl JT, Lønborg J, Fuchs A et al. Assessment of left atrial volume and function: a comparative study between echocardiography, magnetic resonance imaging and multi slice computed tomography. Int J Cardiovasc Imaging 2012;28:1061-71.

12. Dell'Aquila AM, Mastrobuoni S, Bastarrika G et al. Bicaval versus standard technique in orthotopic heart transplant: assessment of atrial performance at magnetic resonance and transthoracic echocardiography. Interact Cardiovasc Thorac Surg 2012;14:457-62.

13. Zhu S, Sun W, Qiao W et al. Real time three-dimensional echocardiographic quantification of left atrial volume in orthotopic heart transplant recipients: Comparisons with cardiac magnetic resonance imaging. Echocardiography 2020;37:1243-1250.

14. Mor-Avi V, Yodwut C, Jenkins C et al. Real-time 3D echocardiographic quantification of left atrial volume: multicenter study for validation with CMR. JACC Cardiovasc Imaging 2012;5:769-77.

15. Perez de Isla L, Feltes G, Moreno J et al. Quantification of left atrial volumes using three-dimensional wall motion tracking echocardiographic technology: comparison with cardiac magnetic resonance. Eur Heart J Cardiovasc Imaging 2014;15:793-9.

16. Ramos JG, Fyrdahl A, Wieslander B et al. Comprehensive Cardiovascular Magnetic Resonance Diastolic Dysfunction Grading Shows Very Good Agreement Compared With Echocardiography. JACC Cardiovasc Imaging 2020;13:2530-2542.

17. Florescu DR, Badano LP, Tomaselli M et al. Automated left atrial volume measurement by two-dimensional speckle-tracking echocardiography: feasibility, accuracy, and reproducibility. Eur Heart J Cardiovasc Imaging 2021;23:85-94.

18. Mor-Avi V, Yodwut C, Jenkins C et al. Real-Time 3D Echocardiographic Quantification of Left Atrial Volume: Multicenter Study for Validation With CMR. JACC: Cardiovascular Imaging 2012;5:769-777.
